# Update on HIV in Western Europe

**DOI:** 10.1007/s11904-014-0198-8

**Published:** 2014-03-22

**Authors:** Fumiyo Nakagawa, Andrew N. Phillips, Jens D. Lundgren

**Affiliations:** 1 Research Department of Infection and Population Health, UCL Royal Free Campus, Rowland Hill Street, London, NW3 2PF UK; 2 Copenhagen HIV Programme (CHIP), Department of Infectious Disease (8632), Rigshospitalet, University of Copenhagen, Blegdamsvej 9, 2100 Copenhagen Ø, Denmark

**Keywords:** HIV infection, HIV/AIDS, HIV, Western Europe, Global epidemic, Europe, HIV testing rates, Antiretroviral therapy, State of the epidemic

## Abstract

HIV infection in Western Europe is mainly concentrated among men who have sex with men, heterosexuals who acquired HIV from sub-Saharan African countries, and in people who inject drugs. The rate of newly diagnosed cases of HIV has remained roughly stable since 2004 whereas the number of people living with HIV has slowly increased due to new infections and the success of antiretroviral therapy in prolonging life. An ageing population is gradually emerging that will require additional care. There are large differences across countries in HIV testing rates, proportions of people who present to care with low CD4+ cell counts, accessibility to treatment and care, and rates of retention once in care. Improved collection of HIV surveillance data will benefit countries and help to understand their epidemic better. However, social inequalities experienced by people with HIV still remain in some regions and urgently need to be addressed.

## Introduction

HIV infection has had a large impact in Western European countries over the last 35 years of the pandemic and continues to affect growing numbers of people. In this review, we summarise key issues and highlight recent articles surrounding the evolution of the HIV epidemic in Western Europe. We focus on the 23 countries found in the West region of the World Health Organisation (WHO) European region (Table 1).

**Table 1:** Data from 2012 on the HIV epidemic in Western Europe: number of HIV diagnoses, estimated number of people living with HIV and disability-adjusted life years (DALYs) attributable to HIV/AIDS

Country	Country population as of 2013 (per million) [48]	Number of HIV diagnoses in 2012 [6]		UNAIDS estimate^1^ of the number of people living with HIV in 2012 [1]		Number of DALYs^2^ attributable to HIV/AIDS (in thousands) [5••]
		N	Rate (per 100,000 population)	Lower estimate	Upper estimate	
Andorra	0.09	2	2.4	–	–	0.0
Austria	8.2	306	3.6	13,000	25,000	3.8
Belgium	10.4	1227	11.1	16,000	26,000	5.3
Denmark	5.6	201	3.6	5500	7500	2.8
Finland	5.3	156	2.9	2600	3600	1.2
France	66.0	4066	6.2	120,000	180,000	48.9
Germany	81.1	2593	3.6	62,000	78,000	29.2
Greece	10.8	1059	9.4	9300	13,000	26.7
Iceland	0.3	219	2.2	<500	<1000	0.4
Ireland	4.8	339	7.4	6300	10,000	1.7
Israel	7.7	487	6.4	6700	11,000	3.2
Italy	61.5	3898	6.4	110,000	140,000	55.3
Luxembourg	0.5	54	10.3	<1000	1100	0.3
Malta	0.4	30	7.2	<500	<500	0.1
Monaco	0.03	0	0	–	–	–
Netherlands	16.8	976	5.8	20,000	34,000	5.2
Norway	4.7	242	4.9	3600	6300	1.2
Portugal	10.8	721	7.0	38,000	62,000	42.8
San Marino	0.03	5	15.5	–	–	–
Spain	47.4	3210	8.5	140,000	170,000	56.2
Sweden	9.1	363	3.8	7200	13,000	1.9
Switzerland	8.0	643	8.1	16,000	27,000	6.8
United Kingdom	63.4	6358	10.3	76,000	120,000	22.2

1) A “best” estimate of the number of people living with HIV in 2012 by country is not given by UNAIDS. 2) DALYs are calculated as the summation of YLLs (years of life lost due to premature mortality) and YLDs (years lived with disability). Disability weights used to calculate YLDs in reference [5••] were: 0.339 for HIV disease resulting in mycobacterial infection, 0.051 for HIV pre-AIDS asymptomatic, 0.221 for HIV pre-AIDS symptomatic, 0.053 for AIDS with antiretrovirals and 0.547 for AIDS without antiretrovirals

### Current State of the Epidemic

The Joint United Nations Programme on HIV/AIDS (UNAIDS) estimates that 860,000 [800,000–930,000] adults were living with HIV in this area of Europe by the end of 2012 [1]. This figure is estimated to have been 360,000 [320,000–400,000] and 570,000 [520,000–600,000] in 1990 and 2000 respectively [1]. UNAIDS estimates are calculated using the Spectrum/Estimation and Projection Package (EPP) software and require country-specific HIV surveillance and survey data [2, 3]. The increasing trend in number of people living with HIV in Europe is a combined result of continued new infections and people living longer because of the success of antiretroviral therapy (ART) [4]. Mortality rates in people with HIV have declined substantially, with most countries in Western Europe having observed at least a 50 % decline from the peak in the mid-1990s to present [5••]. Disability-adjusted life years (DALYs) are used to measure the overall burden of a disease and can be interpreted as the years of potential life lost, due to premature death, disability or ill-health. DALYs are calculated by summing the years of life lost due to premature mortality and the years lived with disability. Estimates of DALYs for HIV in Western Europe are significantly lower than any other region in the world, in which no country has greater than 60,000 DALYs attributed to HIV (Table 1). This compares with 11,915,000 DALYs in South Africa where HIV is the leading cause of DALYs, and 588,000 DALYs in the United States [5••].

**Fig. 1:**
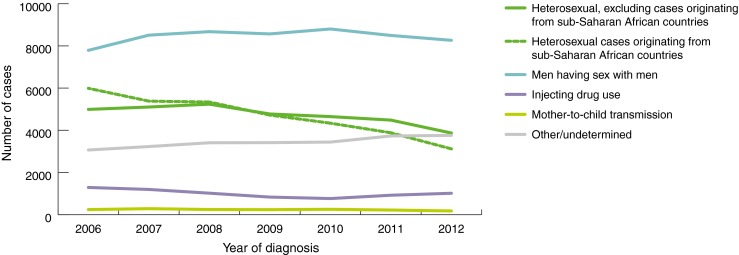
Trends of reported HIV diagnoses by mode of transmission and year of diagnosis in Western Europe. Data not included from Italy and Spain as population coverage of HIV surveillance has increased during this time period. Figure from: European Centre for Disease Prevention and Control/WHO Regional Office for Europe: HIV/AIDS surveillance in Europe 2012 [6]

In 2012, there were 27,315 newly diagnosed cases of HIV reported in Western Europe [6]. This translates to a rate of 6.6 newly diagnosed cases of HIV reported per 100,000 population. The rates of newly diagnosed cases have remained stable since 2004 (all countries except Monaco have been reporting data since 2004 to the European Centre for Disease Prevention and Control, ECDC), with an average of between 6.5 and 7.8 cases per 100,000 each year. Trends over time in this rate however, differ substantially by mode of HIV transmission (Fig. 1) among countries where this has been routinely collected since 2004.

Countries in Western Europe largely have concentrated HIV epidemics, that is, epidemics which are concentrated among sub-populations, particularly men who have sex with men (MSM) and people who inject drugs (PWID). The number of reported HIV diagnoses per year has declined in most transmission groups, including among PWID, cases of mother-to-child transmission (MTCT) and heterosexually acquired cases. Worryingly, a slow but steady increase has been observed in cases among MSM, largely due to rises in number of reported cases in the UK, Belgium, Germany and Israel. The UK contributes around a third of all reported diagnoses among MSM.

The reported data suggest that the HIV epidemic in Western Europe is being fuelled mainly by sexual transmission. In 2012, the distribution of new reported HIV diagnoses by mode of transmission was as follows: sex between men 41.7 %, heterosexual sex 35.3 %, injecting drug use 5.1 %, MTCT 0.7 % and unknown 17.2 %. For every one female diagnosed with HIV, there were 3.1 males diagnosed. Among reported cases in 2012, 9.8 % were in people aged 15 to 24 years old and 16.7 % of cases in people over the age of 50 [6].

## Surveillance of the Epidemic

Knowledge and understanding of epidemics, including monitoring of epidemiological trends and patterns is crucial to inform public health interventions and policies. UNAIDS and WHO have recently published new comprehensive HIV surveillance guidelines to reflect the changing situation, notably wider access to antiretroviral therapy (ART) and prevention of mother-to-child transmission (PMTCT) approaches [7]. On the European level, surveillance for HIV infection has been conducted jointly by ECDC and WHO Regional Office for Europe (WHO/E) since 2007 [8] and by EuroHIV previously [9]. Countries are requested to report each year, the number of HIV diagnoses, AIDS diagnoses, CD4 cell + count (CD4 count) at diagnosis and number of HIV tests performed, to the ECDC through The European Surveillance System (TESSy).

Reports on the number of HIV and AIDS diagnoses, known as ‘case reporting’, form the backbone of HIV surveillance in Europe. Most countries in Western Europe now have nationally established HIV/AIDS case reporting systems (Italy reported national-level data for the first time in 2012 and Spain report aggregate regional-level data, for which coverage has been gradually increasing over time). Many countries also conduct prevalence surveys and cross-sectional qualitative surveys on a regular basis to estimate HIV prevalence and indicators of transmission risk behaviours in key sub-populations [10, 11]. Second generation surveillance of HIV includes surveillance of risk behaviour and surveillance of sexually transmitted infections in addition to case reporting [7]. HIV antibody assay-based approaches to estimate whether an infection was acquired recently (4–12 months) are increasingly used to estimate HIV incidence [12, 13•, 14]. In addition, the SPREAD surveillance programme monitors levels of transmitted drug resistance in newly diagnosed treatment-naïve individuals across Europe [15].

Information obtained through surveillance can contribute to producing estimates of the numbers of people living with HIV and equally importantly, the numbers of people infected but not yet diagnosed. Although the quality and quantity of data collected through surveillance by countries in Western Europe have improved over time, there still remain tricky issues over possible under- and delayed-reporting of cases. One modelling study in 2008 estimated that 35 % of HIV-positive individuals were thought to be undiagnosed in the whole of Western Europe [16]. As yet, relatively few countries have carried out any modelling work to estimate the size of the undiagnosed population in their own countries.

## HIV Care Cascade

The first stage of the spectrum of engagement in HIV care, otherwise commonly referred to as the ‘HIV care cascade’, is the proportion of people with HIV who are aware of their diagnosis status [17]. The subsequent stages are: proportion linked to HIV care, proportion retained in HIV care, proportion receiving ART, and then the proportion with viral suppression. The care cascade is a useful concept to quantify the success of HIV care services and access to ART. Increasing the proportion of HIV-positive people who are on ART with viral suppression is an important aim for HIV prevention, as those with viral suppression are unlikely to transmit HIV [18, 19]. It is encouraging to see that many countries have started to provide such figures, with many reporting that approximately 60 to 70 % of diagnosed HIV-infected individuals are thought to have an undetectable viral load [20–22].

### Testing

A reduction in the proportion of people with HIV who are undiagnosed will be primarily achieved through expansion in coverage and frequency of HIV testing. Even in Western Europe, where ART is widely and generally freely available to all those diagnosed and in need, HIV testing rates vary substantially from 1.9 (Greece) to 119.5 (San Marino) tests performed per 1000 population (where data are collected and excluding unlinked anonymous testing and testing of blood donations) [6]. As a large proportion of transmissions are thought to originate from individuals who are not yet diagnosed [23, 24], earlier and more frequent testing is paramount to benefit, both on an individual- and population-level. In particular, the HIV in Europe Initiative (www.hiveurope.eu) has been successful in promoting optimal HIV testing and earlier care in Europe [25]. Delineation of persons presenting late for care have identified that many were in contact with the health system several times before being diagnosed and hence increased provided-initiated testing is likely to be helpful. The HIV Indicator Diseases across Europe Study (HIDES) was a result of the HIV in Europe 2007 Conference, with the aim to improve targeted testing for people most likely to be infected with HIV [26]. The study authors found that it would be cost-effective to offer an HIV test to anyone presenting with one of eight indicator conditions (sexually transmitted infection, lymphoma, cervical or anal cancer/dysplasia, herpes zoster, hepatitis B/C, mononucleosis-like illness, unexplained leukocytopenia/thrombocytopenia and seborrheic dermatitis/exanthema) [27•].

Although many European countries already have national guidelines for HIV testing [28], ECDC has also issued an evidence-based guidance for testing programmes to help inform recommendations and scale up testing further [29]. Access to testing however seems to vary widely across healthcare settings in Europe: although most countries offer HIV tests in HIV clinics, sexual health clinics, hospitals and antenatal settings, tests are less available through general practitioners, prisons and tuberculosis services [28]. HIV testing has also been found to be feasible in emergency departments, community-based centres and through use of laboratory-based testing of oral fluid, where each have their own benefits and difficulties in implementation [30–33, 34•]. A systematic review assessing barriers to testing in Europe found that there are barriers concerning patients, healthcare providers and at the level of institutions, which include perceived low-risk, fear, accessibility, as well as lack of financial and human resources [35]. These need to be addressed to ensure earlier diagnosis and wide access to care to prevent excess morbidity and mortality. Nonetheless, there is momentum to normalise HIV testing, by means of routine testing such as wider use of opt-out testing, including use of rapid testing, self-sampling (where the sample is posted for the test to be performed), and self-testing (where the person both takes the sample and performs the test themselves) [36, 37].

### Late Presentation

Despite the aforementioned efforts to encourage earlier and more frequent HIV testing and targeting at-risk populations, late presentation (presentation with low CD4 count) is unfortunately not a rarity. A consensus definition has been agreed in order to identify the extent to which it occurs [38, 39]. A common definition of late presentation is important. The term “late” indeed is central, as guidelines across the continent agree that ART should be started before the CD4 count drops below the threshold of 350 cells/mm^3^. Conversely, “late” may not necessarily imply that the person has been infected for several years, since data from seroconverter cohorts suggest that for around 20 % of people, the CD4 count is below 350 cells/mm^3^ by one year from infection [40]. Among adult HIV diagnoses in the EU/EEA (European Union and European Economic Area) region reported to ECDC (56 % completeness in 2011), 49 % were reported as late presentations (CD4 count at diagnosis <350 cells/mm^3^) and 29 % were reported as advanced HIV disease (CD4 count at diagnosis <200 cells/mm^3^) [8]. A large cohort collaboration study found that late presentation in Europe has declined from 57.3 % in 2000 to 51.7 % in 2010/2011; however late presentation was found to be most prevalent in heterosexual males, Southern European countries (Greece, Israel, Italy, Portugal and Spain), and people originating from Africa, suggesting key populations and regions for priority interventions [41••].

### Access and Retention in Care

Retention in care includes both linkage to care following a diagnosis and subsequent retention in care. In particular, a clear and established pathway for onward referral for patients who test HIV-positive is intrinsic to achieve good rates of integration into HIV care services for timely ART initiation and attaining viral suppression. Many of the barriers that limit access to and retention in care elsewhere have been reduced and even removed in healthcare systems in Western Europe. Care is generally free and focuses not only to treat HIV infection but also co-infections and co-morbidities, including addiction and psychiatric diseases [42]. There are a minority of settings of concern that do not provide universal access to HIV prevention, treatment and care. The recent change in legislation in Spain preventing illegal immigrants from accessing HIV care is one notable example [43]. However, most people on ART in centres across Western Europe experience continued complete viral control. The level of viral load is the main predictor of HIV transmission risk [44]. Therefore the higher the proportion of people with suppressed viral load, the less chance of transmission of both drug-susceptible and drug-resistant strains. Perhaps largely due to on-going high rates of suppression in Europe, prevalence of transmitted drug resistance has been maintained below 10 % [45].

As life expectancies have improved over time in people living with HIV, we are now used to seeing a situation in Western Europe where a large group of people seen in care are over the age of 50 [46, 47]. The proportion of newly diagnosed cases of HIV in people aged 50 and above has also been gradually increasing each year, up from 11.9 % in 2006 to 16.6 % in 2012 [6]. It will therefore be important to ensure that HIV care services are adapted so that the needs of older people living with HIV are met. A more integrated approach to managing care may be appropriate, which would involve working closer with primary care physicians to manage HIV-related conditions but also non-HIV-related conditions which occur more frequently with older age.

## Men Who Have Sex with Men (MSM)

### Prevalence and Incidence

HIV infection has had a large impact on MSM in Western Europe and it still remains the predominant mode of HIV transmission. Reported prevalence is high, ranging from 0.5 to 17.7 % [49]. The highest prevalences were found in France (17.7 %), Spain (13.1 %), Greece (12.7 %) and Germany (11.5 %). It is important to note here that these data were from various sources, mainly prevalence surveys, conducted in each country separately and may differ greatly by risk group definition, definition of a man who has sex with men, representativeness of the sample and sample size.

The UK, France, Netherlands, Germany, Denmark and Spain are examples of countries that have seen a gradual rise in the number of HIV diagnoses reported among MSM [8]. Studies published in the last few years have indicated that these rises are not just a result of higher testing rates and better surveillance systems, but are a result of an increase in transmission by condom-less sex [14, 50–52, 53•]. In many of these new infections, the probable source is thought to be HIV-positive people who are not yet diagnosed. These observations have been seen despite high ART coverage in people with CD4 count <350 cells/mm^3^ [54].

### Risk Behaviour

Results from the European MSM Internet Survey (EMIS) found that men known to be HIV-positive were 2.4-fold more likely to report condom-less sex with a partner of discordant or unknown status (CLS-D) than HIV-negative and untested men [55]. The proportion of men who had CLS-D with any male partner in the preceding 12 months ranged from 25 to 41 %. The study also found that countries in the Central-West and West regions had the lowest levels of CLS-D whereas countries in the South-East regions had the highest levels. Similarly, condom-use at last anal intercourse with a male partner also ranged from 28 to 76 % over 35 countries in Europe, although around half the countries reported proportions between 40 and 60 % [49]. Despite the limitations of the uncertain representativeness of those sampled, and the potential for social desirability bias in reporting, these data seem to indicate that sexual risk behaviour among MSM varies considerably from country to country.

## Heterosexual Transmission

### Migration from Countries with Generalised HIV Epidemics

Migrants from countries with generalised HIV epidemics, mainly from sub-Saharan Africa, represent a large proportion of heterosexually-acquired HIV and AIDS case reports in Western Europe. Although there are large disparities due to former colonial links, healthcare systems and social structures, the general trend is such that countries with large migrant populations in the general population will have large numbers of migrants among those presenting with HIV [56]. The reported number of cases of HIV diagnoses in Western Europe originating from sub-Saharan Africa has nearly halved in the years between 2006 and 2012 (Fig. 1) [6]. In contrast, for example in the UK, there has been a rise in the proportion of diagnoses among people born abroad but who probably acquired their infection in the UK [57].

Of individuals infected through heterosexual contact and diagnosed in 2011, the proportion of reported cases in people originating from a country with a generalised epidemic varied from 5.6 % in Finland to 66.4 % in Ireland, which are much higher than levels seen in Central and Eastern European countries [6]. A major concern is that more than 60 % of migrants from generalised epidemic countries are considered to be late presenters [58] and many probably have a CD4 count below 350 cells/mm^3^ before arrival in Europe. As mentioned earlier, although many countries recognise that migrants and ethnic minorities are particularly vulnerable to HIV [59], testing and care are not universally available in all settings, particularly for those with uncertain or illegal migrant status.

### Migration from East to West

The effect of migration from Eastern to Western Europe is unclear, but absolute numbers of European migrants diagnosed with HIV are small to date [56]. The exceptions are for countries with close borders to countries in the Eastern Europe and Central Asia region where the epidemic has around the turn of the millennium been mainly driven by injecting drug use, but more recently mixed with heterosexual and some MSM transmission [60, 61].

## Injecting Drug Use

### Importance of Harm-Reduction Services

Across Western Europe, there have been low rates of new infections among PWID due to the wide implementation and success of harm-reduction policies and services [6]. Where data are available, it seems that all countries in Western Europe provide needle and syringe exchange programmes, opioid substitution treatment (OST) and ART for PWID, which is much higher than the average global coverage [62].

However, a number of European countries reported a rise in the number of new HIV diagnoses among PWID in 2011, most notably Greece and Romania [63•]. Both countries reported a ten-fold rise; in Greece, the number of HIV diagnoses increased from 22 cases in 2010 to 245 cases in 2011 and similarly in Romania, from nine cases in 2010 to 108 cases in 2011 [8]. Greece attributes the outbreak to the shortage of needle and syringe exchange programmes and long waiting times for access to OST prior to 2011 [64]. In Romania, evidence from behavioural surveillance surveys conducted in PWID suggested changes in drug-use patterns (amphetamine-type stimulants, associated with more frequent injections and increased sharing of injecting equipment, became more common than heroin) [64]. However similarly to the situation in Greece, access to harm reduction services also declined alongside due to the withdrawal of international programs and funding support [63•, 64]. These recent outbreaks of HIV transmission among PWID serve as a reminder that these policies are fragile and vulnerable in countries affected by economic constraints, and stress the need for continuing public health and preventative services.

### Co-Infection with Hepatitis C

PWID are at the highest risk of being co-infected with hepatitis C virus (HCV). Of patients in a Europe-wide cohort 78 % who were HCV-positive had acquired HIV-1 via injecting drug use and most likely acquired HCV through this route too [65]. For people with HCV, co-infection with HIV is known to be strongly associated with poorer prognosis with an increased risk of developing liver disease and HCV-related mortality [66]. Expanded access to HCV diagnosis and treatment will therefore be important in conjunction with harm-reduction and HIV-related programs to control the evolving epidemic.

## Mother-to-Child Transmission (MTCT)

The probability of a child with an HIV-positive mother being born with HIV have now reduced to approximately 1 to 2 % in Western Europe, due to effective preventative interventions including universal antenatal testing, caesarean sections, formula feeding and particularly through use of ART [67, 68]. However over the last decade, it has been shown that 40 % of MTCT are due to insufficient antenatal ART for the women, even though half of them were diagnosed pre-conception [69]. It appears that there remain missed opportunities for prevention of MTCT which may be improved by better engagement and retention in care and by providing support for women before, during and after pregnancy, to achieve and sustain viral suppression [69, 70].

## Social Inequalities

The HIV epidemic in Western Europe is concentrated in MSM and PWID, but also disproportionately affects other marginalised populations including migrants, sex workers, transgender people and prisoners. HIV-related stigma and discrimination are commonly faced by these populations, acting as a major barrier to the integral care and support that is needed. These barriers can hinder the success of interventions and programmes to provide access to prevention schemes, HIV testing, linkage to and retention in care, and use of ART [35, 71–73]. We highlight the recent events in Greece as a regretful example, where police detained, tested and then publicised people’s HIV status [74]. These actions, as a consequence of the change in law, have and will continue to damage and undo all the benefits which have taken place so far to combat the epidemic.

The Euro HIV index 2009 was a study conducted to assess the quality of HIV policies and services and the social inequalities present in 29 European countries. The study authors concluded that the countries rated as having the best HIV care were Luxembourg, Malta, Switzerland, Finland and the Netherlands [75]. These countries were the ones with the most effective prevention programmes and good access to care, especially for people who are part of marginalised populations. Societal inequalities existed in varying degrees among the countries surveyed, but the notable areas requiring improvements were the stigma and discrimination faced by vulnerable and marginalised populations and lack of universal access to HIV testing, care and treatment.

## Conclusions

Although in Western Europe the absolute number of people affected by HIV is relatively small compared to other areas such as sub-Saharan Africa and Eastern Europe, it is still a significant public health issue. Fortunately, many countries within this region are resource-rich and have been equipped with solid infrastructures and good quality healthcare systems. Consequently, Western Europe leads globally in terms of the accessibility and standard of HIV care, but even within this region, large differences are seen, as found in the Euro HIV Index 2009. One issue that the study highlighted was the lack of good epidemiological data. Understanding the individual country epidemics, by means of collecting good quality surveillance data, is crucial to be able to provide the appropriate services and programs that are needed in these settings [76•]. Estimation of the number of people living with HIV and the size of the undiagnosed proportion would therefore be very informative for people working in public health and policy makers.

With no evidence of a decline in HIV incidence in Western Europe, it will be important to plan for and provide the appropriate HIV services for years to come. A major challenge which is faced by many countries already is taking care of the ageing population, including people newly diagnosed but also those who have lived with HIV for decades and are now reaching ages where the risk of comorbidities are much greater. Lastly, there is still a vital need to address the barriers which exist that prevent universal access to HIV prevention services, treatment and care.
